# SMAD4 Limits PARP1 dependent DNA Repair to Render Pancreatic Cancer Cells Sensitive to Radiotherapy

**DOI:** 10.1038/s41419-024-07210-7

**Published:** 2024-11-11

**Authors:** Yang Wang, Tianyu Yu, Zhangting Zhao, Xiaobing Li, Yiran Song, Yazhi He, Yingqun Zhou, Pu Li, Liwei An, Feng Wang

**Affiliations:** 1 Department of Gastroenterology, Huadong Hospital, Fudan University, 200040 Shanghai, China; 2 Department of Gastroenterology, Shanghai Tenth People’s Hospital, Tongji University School of Medicine, 200072 Shanghai, China; 3 Department of Stomatology, Shanghai Tenth People’s Hospital, Tongji University School of Medicine, 200072 Shanghai, China; 4 Center for Molecular Recognition and Biosensing, School of Life Sciences, Shanghai University, 200444 Shanghai, China; 5 Department of Pediatrics, Ruijin Hospital, Shanghai Jiao Tong University School of Medicine, 200025 Shanghai, China

**Keywords:** Radiotherapy, Cancer therapeutic resistance

## Abstract

Dysregulation of SMAD4 (i.e. somatic mutation) is strongly associated with poor pancreatic ductal adenocarcinoma (PDAC) prognosis, yet the molecular mechanisms remain underlying this relationship obscure. Previously, we discovered that *SMAD4* mutation renders pancreatic cancer resistant to radiotherapy via promotion of autophagy. In the current work, we observed a downregulation of the protein level of SMAD4 in PDAC as compared with adjacent normal tissue, and that such SMAD4^low^ PDAC failed to benefit from chemotherapy. Furthermore, we observed that SMAD4 depletion dramatically enhanced DNA repair capacity in response to irradiation (IR) or a radiomimetic chemical. Interestingly, we found the radiomimetic chemical having induced a robust translocation of SMAD4 into the nucleus, where a direct interaction was shown to occur between the MH1 domain of SMAD4 and the DBD domain of PARP1. Functionally, the SMAD4-PARP1 interaction was found to perturb the recruitment of PARP1 to DNA damage sites. Accordingly, the combination of olaparib and radiotherapy was indicated in vivo and in vitro to specifically reduce the growth of SMAD4-deficient PDAC by attenuating PARP1 activity. Collectively, our results revealed a novel molecular mechanism for the involvement of the SMAD4-PARP1 interaction in DNA repair with a vital role in radiotherapy response in PDAC. Based on our set of findings, our findings offer a new combined therapeutic strategy for SMAD4 deficient PDAC that can significantly reduce pancreatic cancer radiotherapy resistance.

## Introduction

Pancreatic ductal adenocarcinoma (PDAC) is characterized by late detection and resistance to therapy, culminating in a dismal five-year survival rate below 10% [1, 2]. Predominantly mutated genes including the oncogene *KRAS*, along with tumor suppressors *CDKN2A*, *TP53* and *SMAD4*, are most identified in PDAC. SMAD4 loss, observed in 55% of patients, is associated with increased radiotherapy resistance and a poorer prognosis in PDAC [3, 4]. Our prior work has demonstrated that SMAD4 deletion exacerbates radiotherapy resistance by enhancing reactive oxygen species (ROS) production and radiation-induced autophagy [5]. Tailored application of specified inhibitors might address tumor progression in patients carrying these mutations. For instance, preclinical studies have shown that inhibitors of CDK4/6 can curb tumor growth in KRAS-mutant PDAC models [6]. However, no effective method to boost radiotherapy sensitivity in patients displaying SMAD4 deficiency has yet been established.

Radiotherapy targets tumors by inducing DNA damage on the genome for apoptosis. Tumor cells counteract this therapy through sophisticated DNA damage response (DDR) mechanisms that detect and repair DNA lesions [7, 8]. Poly (ADP-ribose) polymerase 1 (PARP1), a key component of the PARP family, plays a pivotal role in DDR. Upon DNA damage, PARP1 rapidly localizes to the site of damage and initiates the formation of poly (ADP-ribose) (PAR) chains, which signal the recruitment of additional DDR proteins [9, 10]. While inhibitors of PARP have been approved for BRCA-mutated cancers, exploiting synthetic lethality in DNA repair pathways [11, 12], only 5-10% of pancreatic cancer patients possess BRCA1 or BRCA2 mutations [1, 13], limiting the applicability of PARP inhibitors in pancreatic cancer treatment.

SMAD4 serves as a crucial transcription factor in the TGF-β signaling pathway. When the TGF-β signaling pathway is activated, SMAD2 and SMAD3 are first phosphorylated, after which they bind to SMAD4 to form a heterodimeric complex and then translocate to the nucleus to either activate or repress target genes [14]. In the current study, we have found a radiomimetic chemical that can induce a robust nuclear translocation of SMAD4 in a non-TGF-β-dependent manner. In the nucleus, SMAD4 was determined to interact with the DBD domain of PARP1 through its MH1 domain, inhibiting recruitment of PARP1 to DNA damage sites and thereby reducing DNA repair efficiency. Furthermore, in our experiments, combining the PARP inhibitor olaparib with radiotherapy significantly mitigated radiotherapy resistance in SMAD4-deficient PDAC. Our study also suggested a mechanism by which dysregulated PARP1 activity can contribute to radiotherapy resistance in SMAD4-deficient PDAC, providing a theoretical basis for the use of PARP inhibitors for enhancing radiotherapy efficacy in SMAD4-deficient PDAC patients.

## Materials and Methods

### Cell lines and culture conditions

Cell lines Panc-1, Bxpc-3, and HEK293FT were purchased from the American Type Culture Collection (ATCC, USA). The Panc-1 and HEK293FT cells were cultured in Dulbecco’s modified Eagle’s medium (DMEM, Gibco, USA) enriched with 10% fetal bovine serum (FBS, Gibco, USA) and 100 μg/mL of penicillin/streptomycin (Gibco, USA). The Bxpc-3 cells were cultured in Roswell Park Memorial Institute 1640 medium (RPMI 1640, Gibco, USA) supplemented with 10% FBS and 100 μg/mL of penicillin/streptomycin. All cells were continuously maintained in an environment of 5% CO_2_ at 37 °C.

### Plasmids

SMAD4 and PARP1, along with their mutants, were subcloned into a modified pCDH vector featuring an N-terminal 3xFlag tag. These constructs were also introduced into pLV-EGFP tagged with GFP and pLV vectors tagged with HA, respectively. All resulting plasmids were confirmed by performing DNA sequencing.

### Transfection, lentivirus packaging, and infection

To facilitate transfections, plasmid constructs were pre-incubated with polyethyleneimine (PEI, Cat# 23966-1, Polysciences, USA) at a ratio of 1:4 in Opti-MEM (Thermo Fisher Scientific, USA) for 15 min at room temperature before being administered dropwise into the cell culture medium. For the production of lentiviral particles, lentiviral-based constructs were co-transfected with the packaging plasmids psPAX2 and pMD2.G into HEK293FT cells at a mass ratio of 4:3:1. The resulting supernatants were collected at 24, 48, and 72 h after transfection, filtered through a 0.45 μm pore size filter, and used to transduce target cells in the presence of 10 μg/mL polybrene (Cat# 28728-55-4, Sigma-Aldrich, USA). Additionally, vectors carrying human SMAD4-specific shRNA with the target sequence 5’-GGTGTGCAGTTGGAATGTA-3’, along with non-targeting controls, were obtained from Genechem (China).

### Antibodies and chemical agents

The antibodies used in this study are listed in Supplementary table. Chemical agents used included the radiomimetic drug NCS (Cat# 9014-02-2, Sigma-Aldrich, USA) and the PARP inhibitor olaparib (Cat# S1060, Selleck, USA).

### Immunoprecipitation and western blot analysis

For the co-immunoprecipitation (co-IP) assays, cells were lysed using NETN buffer (20 mM Tris-HCl, pH 8.0, 100 mM NaCl, 1 mM EDTA, and 0.5% NP-40) for 30 min at 4 °C. Lysates were then centrifuged at 12,000 rpm for 15 min at 4 °C. The resulting clarified supernatants were incubated overnight at 4 °C with rotation using anti-DYKDDDDK affinity beads (Cat# SA042025, Smart-Lifesciences, China), anti-GFP affinity beads (Cat# SA070025, Smart-Lifesciences, China), streptavidin beads (Cat# SA021100, Smart-Lifesciences, China), or Protein A/G PLUS Agarose beads (Cat# sc-2003, Santa Cruz Biotechnology, USA) conjugated with the appropriate antibodies. Post-incubation, beads were washed thrice with NETN buffer and eluted by boiling them in 1x SDS loading buffer. To minimize interference from immunoglobulin light/heavy chains, specific horseradish peroxidase (HRP)-conjugated secondary antibodies (Cat# M21008, Abmart, China) were used at a 1:500 dilution.

### Immunofluorescence staining

To carry out immunofluorescence staining, first the target cells were seeded onto glass-bottom dishes (Cat# D29-20-1.5-N, Cellvis, USA), and after 24 h washed thrice with phosphate-buffered saline (PBS) and fixed with 1 ml of 4% paraformaldehyde at room temperature for 10 min. The cells were then permeabilized with 0.5% Triton X-100 (Cat# A600198, Sangon Biotech, China) for one minute. The permeabilized cells were subjected to three additional washes with PBS, and then their non-specific binding sites were blocked using 3% bovine serum albumin (BSA) for 30 min. The cells were subsequently incubated for two hours at room temperature with primary antibodies, diluted in primary antibody dilution buffer (Cat# P0023A, Beyotime Biotechnology, China). Following the primary antibody incubation, the cells were washed thrice with PBS and incubated for one hour at room temperature in the dark with fluorochrome-conjugated secondary antibodies, also diluted in primary antibody dilution buffer. The resulting cells were subjected to three more washes with PBS, and then to nuclear staining using 0.2 μg/mL DAPI for five minutes, followed by a final wash with PBS. Images of the cells were acquired using a confocal microscope (ZEISS, Germany).

### Immunohistochemistry (IHC) staining

Tissue microarray sections were constructed and provided by PowerX-Bio (China), and then subjected to immunohistochemistry (IHC) staining by incubating them with anti-SMAD4 antibody (Cat# ab40759, Abcam, UK) and anti-PARP1 antibody (Cat# ab191217, Abcam, UK). The expression levels of these two markers were qualitatively assessed from IHC staining images by three independent pathologists and categorized into low (-) and high (+) expression groups. Also, comprehensive clinical data from the patients providing the tissue samples were meticulously collected.

### Mass spectrometry

HEK293FT cells transfected with specific plasmids were lysed using NETN buffer. The resulting supernatants were incubated with anti-streptavidin beads at 4 °C overnight. Afterward, the beads were washed thrice with NETN buffer and then subjected to mass spectrometric (MS) analysis by OE Biotech (China).

### Colony formation assay

Cells were seeded at a density of 1000 per well into six-well plates in triplicate. After a 48 h incubation, they were exposed to ionizing radiation (IR) and subsequently maintained in samples of medium supplemented with different concentrations of olaparib. After 12 days, the colonies were gently washed with PBS and fixed with 1 ml of 4% paraformaldehyde at room temperature for 10 min. Then added crystal violet (C0121, Beyotime Biotechnology, China) for 10 min. The visible colonies were counted, and the survival fraction was calculated based on these counts.

### Laser micro-irradiation and live-cell imaging

Cells were cultured on glass-bottom dishes (Cat# D29-20-1.5-N, Cellvis, USA) overnight. They were then transfected with GFP-PARP1 and treated with BrdU (Cat# B9285, Sigma-Aldrich, USA) for 24 h. Subsequently, the cells were incubated with Hoechst 33342 (Cat# 6249, Thermo Fisher Scientific, USA) for 15 min and washed thrice with fresh medium. For live-cell imaging, the cells were transferred to a cell culture chamber, maintained at 37 °C and 5% CO_2_, on an inverted microscope (Nikon, Japan). Images were acquired at 30-second intervals over a period of 10 min and exported as TIFF files.

### In vivo mouse model assays

Male BALB/c nude mice, aged 3–4 weeks, were purchased from Shanghai Model Organisms Center (China). All mice were housed in a standard animal facility at 22 °C, with access to sterile standard food and water. The care and use of the animals were approved by the ethical review board at Shanghai Tenth People’s Hospital (under references SHDSYY-2023-2532). All methods were performed in accordance with the relevant guidelines and regulations at Tongji University and Shanghai Tenth People’s Hospital. For tumor induction, 5 × 10^6^ cells were injected subcutaneously into the right flank of each mouse. Tumor size was monitored every three days and tumor volume was estimated as 0.5 × length × width × width. After four weeks, the mice were sacrificed, and tumors were dissected and weighed.

### Statistics

Data for the statistical analysis were derived from at least three independent experiments and were analyzed with GraphPad Prism 8.0 statistical software. Student’s t-test was employed to assess statistical significance to compare two variables. Survival outcomes were analyzed using the log-rank test. Values of *P* < 0.05 were considered to indicate for statistical significance. Survival curves were calculated according to the Kaplan–Meier method; survival analysis was performed using the log-rank test.

## Results

### SMAD4 deficiency endows PDAC with radioresistance

**Fig. 1:**
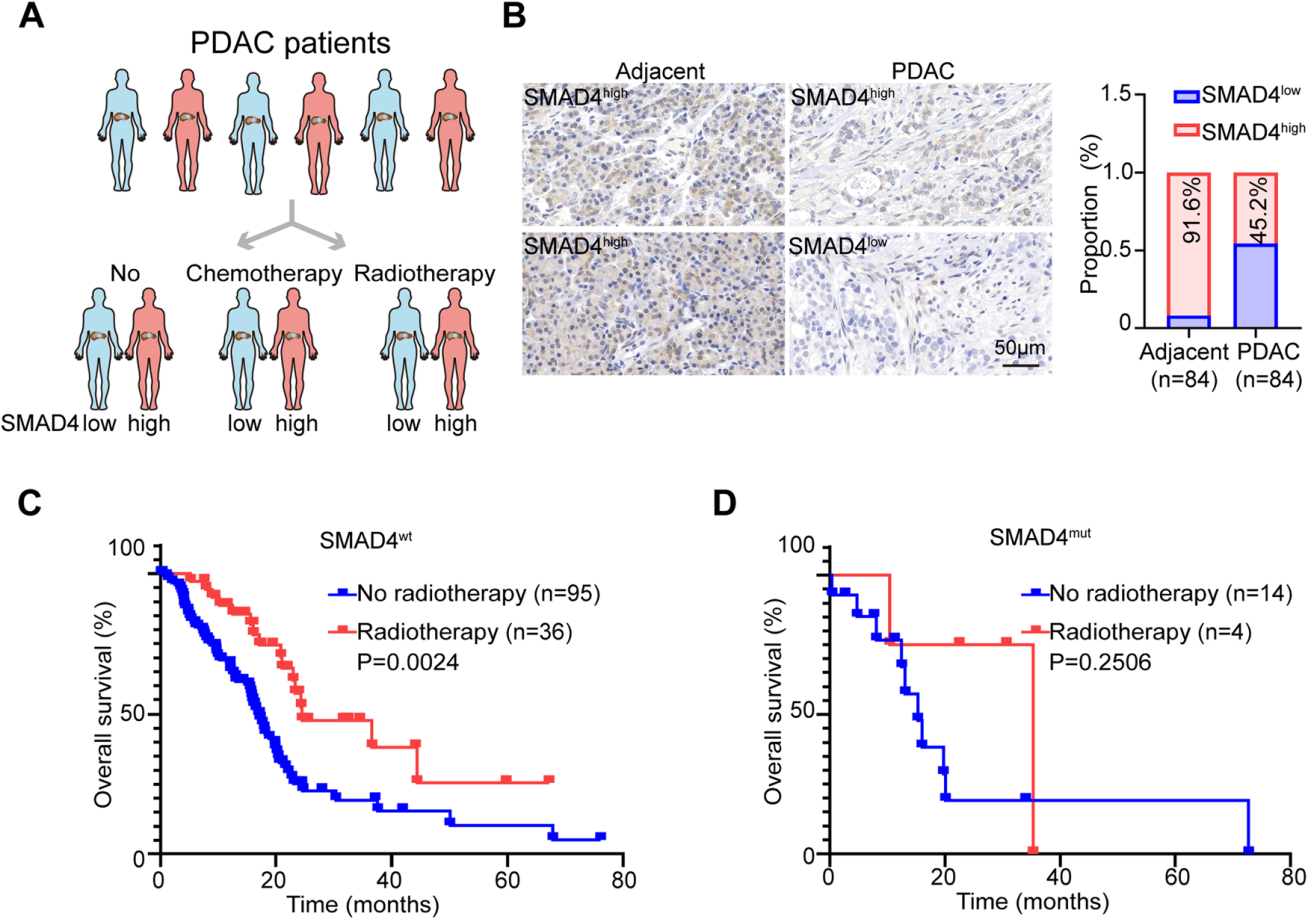
SMAD4 mutation confers resistance to radiotherapy in PDAC. **A** Schematic diagram illustrating the treatment groups of PDAC patients. **B** Representative IHC images showing the levels of SMAD4 derived from 84 adjacent normal tissues and tumor tissues from PDAC patients (scale bar, 50 µm). Based on the SMAD4 protein staining level, each group was classified as low or high expression level. **C** Plots showing an association of SMAD4 with a favorable OS in SMAD4^wt^ PDAC patients after radiotherapy treatment. **D** Plots showing a lack of correlation of SMAD4 with OS in SMAD4^mut^ PDAC patients with or without radiotherapy treatment.

To investigate the clinical implications of the SMAD4 status on radiotherapy resistance, we conducted IHC analyses of SMAD4 in tissue microarrays from 86 pancreatic ductal adenocarcinoma (PDAC) patients, comparing tumor tissues with adjacent normal tissues (Fig. 1A). We observed a downregulation of SMAD4 in tumor tissues relative to paired paraneoplastic samples in 84 PDAC patients (Fig. 1B), and 2 tumor tissues were not counted due to the absence of paired paraneoplastic samples. Additionally, an analysis of 149 PDAC patients from The Cancer Genome Atlas (TCGA) indicated a correlation of SMAD4 mutations with poorer prognosis in patients undergoing radiotherapy: patients with wild-type SMAD4 (SMAD4^wt^) who received radiotherapy showed significantly longer overall survival (OS) compared to those who did not (Fig. 1C); conversely, radiotherapy did not significantly extend OS in patients with mutated SMAD4 (SMAD4^mut^) (Fig. 1D). This pattern was mirrored in chemotherapy outcomes, where SMAD4^wt^ patients benefited from chemotherapy with improved OS, whereas SMAD4^mut^ patients appeared to show no significant benefit (Supplementary Fig. 1A, B).

In addition, no significant differences were found in OS between patients with low SMAD4 expression (SMAD4^low^) and those with high SMAD4 expression (SMAD4^high^) regardless of chemotherapy status (Supplementary Fig. 1C, E). Similarly, disease-free survival (DFS) did not differ significantly between the groups not receiving chemotherapy (Supplementary Fig. 1D). Interestingly, patients with low SMAD4 expression who underwent chemotherapy exhibited significantly shorter DFS compared to those with high expression (Supplementary Fig. 1F). The discrepancies between the findings based on the mutational status of SMAD and those based on expression level may have stemmed from the different methodologies used to assess SMAD4 status—immunohistochemistry (IHC) for expression levels and whole-exome sequencing for mutations. Regardless, these findings suggested a strong association between SMAD4 deficiency and radioresistance in PDAC patients.

### SMAD4 deficiency enhances radiomimetic chemical-induced DNA damage repair

**Fig. 2:**
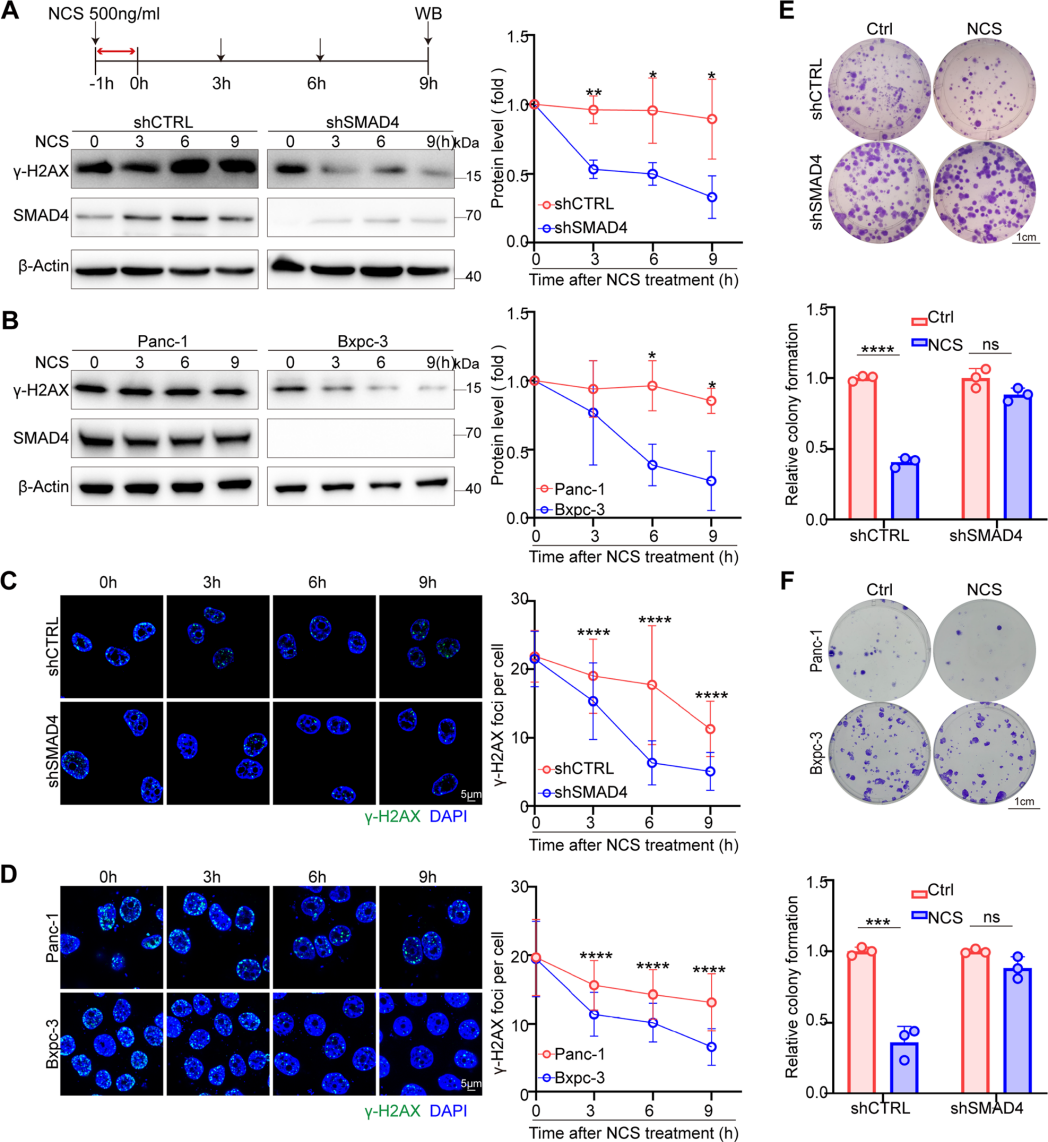
SMAD4 deficiency enhances DNA damage repair. **A** Gels showing SMAD4 having attenuated DNA repair capacity. Cells were exposed to 500 ng/ml NCS for one hour and then collected at the indicated time points after removal of the drug to determine the γ-H2AX protein levels. **B** Gels showing SMAD4 having attenuated DNA repair capacity. Cells were exposed to 500 ng/ml NCS for one hour and then collected at the indicated time points after removal of the drug to determine the γ-H2AX protein levels. **C** Images showing SMAD4 having attenuated DNA repair capacity. Cells were exposed to 500 ng/ml NCS for 1 h and then collected at the indicated time points after removal of the drug to determine the γ-H2AX protein levels. **D** Images showing SMAD4 having attenuated DNA repair capacity. Cells were exposed to 500 ng/ml NCS for 1 h and then collected at the indicated time points after removal of the drug to determine the γ-H2AX protein levels. **E** Clonogenic formation assay to evaluate the viability levels of shCTRL and shSMAD4 cells after they were treated with NCS. **F** Clonogenic formation assay to evaluate the viability levels of Panc-1 and Bxpc-3 cells after they were treated with NCS. *P < 0.05, **P < 0.01, and ***P < 0.001 by unpaired Student’s t-test.

Neocarzinostatin (NCS) is a radiomimetic antibiotic shown to induce DNA damage [15, 16]. To investigate the influence of SMAD4 on radiotherapy sensitivity in PDAC, we examined the effects of SMAD4 deficiency on NCS-induced γ-H2AX expression and foci formation in PDAC cells. Our findings revealed a significant reduction in γ-H2AX expression following NCS-induced DNA damage in SMAD4 knockdown PDAC cells (Fig. 2A, C), suggesting an enhanced DNA repair capability. Consistent with these results, γ-H2AX levels had a more rapid decline over time in the Bxpc-3 cells lacking SMAD4 expression than in Panc-1 cells with intact SMAD4 (Fig. 2B, D), indicating a superior DNA repair capacity. Additionally, a colony formation assay demonstrated that SMAD4 deficiency significantly increased clonogenic survival following NCS-induced DNA damage (Fig. 2E, F). Collectively, these results suggested that SMAD4 deficiency may enhance NCS-induced DNA damage repair and contribute to increased radioresistance in PDAC cells.

### Radiomimetic chemical NCS enhances SMAD4 nuclear translocation and fosters its interaction with PARP1

**Fig. 3:**
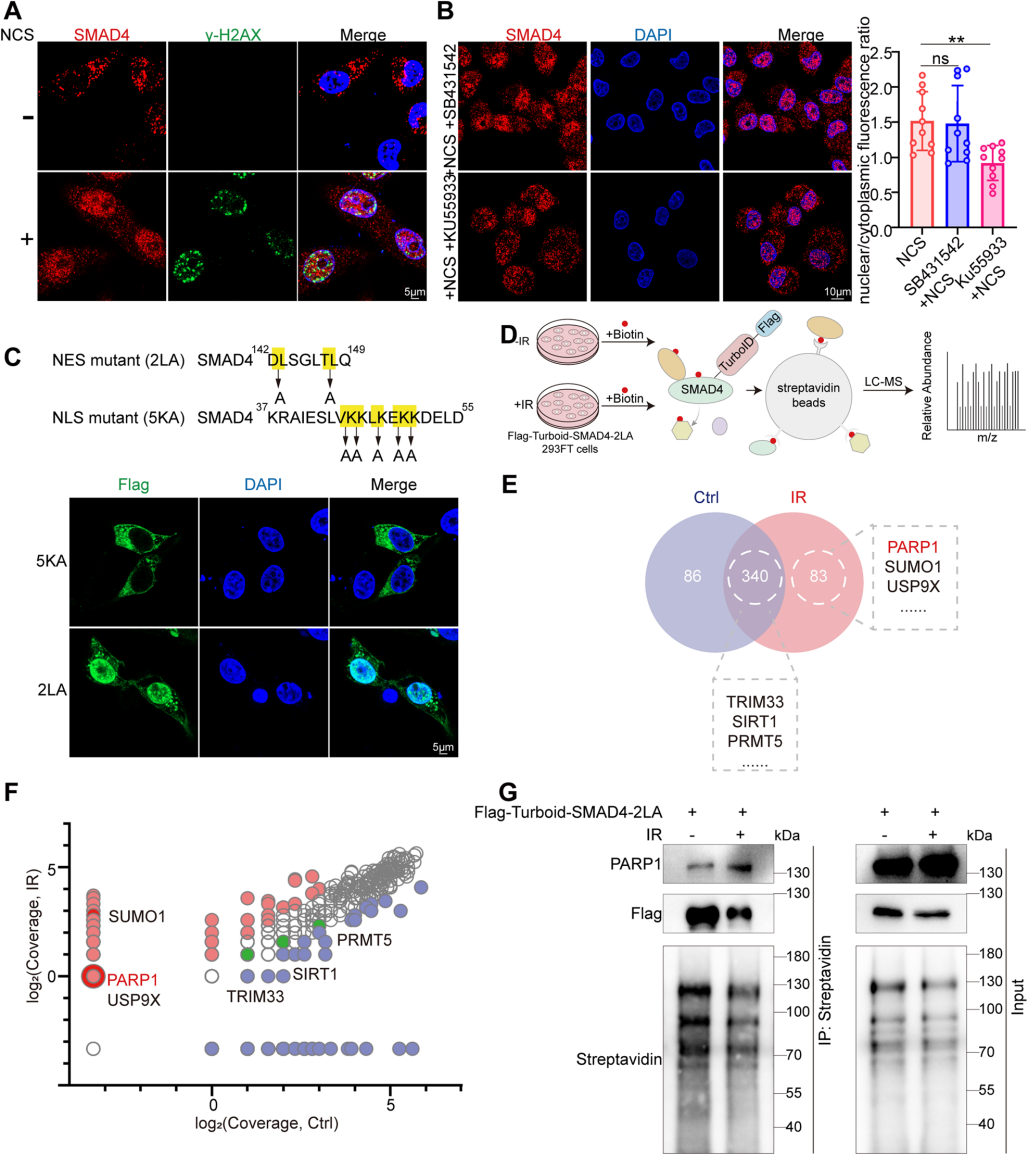
Irradiation enhances SMAD4 nuclear translocation and fosters its interaction with PARP1. **A** Images showing increased SMAD4 protein expression in the nucleus after NCS treatment. Panc-1 cells were exposed to 500 ng/ml NCS for 1 h. **B** Images showing decreased SMAD4 protein expression in the nucleus after NCS and KU55933 treatment. Panc-1 cells were exposed to 10 μM KU55933 for 2 h and then 500 ng/ml NCS for 1 h. **C** Images showing the locations of SMAD4-5KA and SMAD4-2LA mutants. **D** Schematic representation of the TurboID proteomic assay technique used to analyze SMAD4 interactions upon induction of DNA damage. **E** Venn diagram of the proteins interacting with nuclear SMAD4 in the cells subjected to DNA-damaging irradiation (pink) and controls (blue). Number of proteins interacting with SMAD in both types of treatments and the numbers of those interacting only in one case or the other are shown. **F** Results of the TurboID assay identifying of PARP1 as a potential SMAD4-interacting protein. **G** HEK293FT cells transfected with Flag- TurboID-SMAD4-2LA were treated with irradiation and biotin for indicated time points followed by SMAD4 co-IP assay. *P < 0.05, **P < 0.01, and ***P < 0.001 by unpaired Student’s t-test.

SMAD4, which shuttles between the cytoplasm and nucleus, accumulates in the nucleus following TGF-β stimulation [17]. We thus further inquired whether radiotherapy affects SMAD4 nuclear translocation. As shown in Fig. 3A, with the inclusion of NCS triggering DNA damage, more SMAD4 translocated into the nucleus. We then further investigated the regulatory mechanisms of the effects of radiotherapy on SMAD4 entry into the nucleus. It is widely accepted that the protein kinase ataxia telangiectasia mutated (ATM) is a master regulator of double-strand DNA break (DSB) signaling and stress [18]. When including KU55933, an inhibitor of ATM activity, we observed a significant reduction in NCS-induced nuclear translocation of SMAD4 (Fig. 3B). Additionally, we assessed the role of TGF-β signaling in this context by employing SB431542, a TGF-β inhibitor. Interestingly, inhibiting TGF-β signaling in this manner did not significantly affect the NCS-induced nuclear translocation of SMAD4 (Fig. 3B), indicative of the irradiation-induced translocation of SMAD4 being mediated through the ATM pathway and being independent of the TGF-β pathway. These findings highlighted a distinct pathway by which radiotherapy may modulate SMAD4 localization and function.

SMAD4 contains both a canonical nuclear export signal (NES) and a classical nuclear localization signal (NLS) [19]. The SMAD4-2LA mutant, produced by mutating two NES critical leucine residues (Leu-146 and Leu-148) of SMAD4 to alanines, predominantly localized in the nucleus; in contrast, the SMAD4-5KA mutant, produced by mutating NLS key lysine residues, was completely excluded from the nucleus (Fig. 3C). To elucidate the molecular mechanisms through which SMAD4 affects radioresistance in PDAC cells, we conducted mass spectrometry to identify nuclear proteins interacting with the nuclear-localized SMAD4 (Fig. 3D). Post-irradiation, we observed significant changes in the protein profile associated with nuclear SMAD4, with DNA damage repair proteins such as PARP1 and XRCC5 showing increased binding affinity for SMAD4 (Fig. 3E, F and Supplementary Fig. 2A, B). Notably, PARP1, a key protein in DNA damage repair and whose inhibitors are approved for various cancers, was previously shown to demonstrate enhanced interaction with SMAD4 [20]. Therefore, we further performed a co-immunoprecipitation (co-IP) analysis and found that after IR treatment, SMAD4-2LA exhibited increased binding to endogenous PARP1 in HEK293FT cells (Fig. 3G). Additionally, despite the role of SMAD4 as a transcription factor, RNA sequencing analysis indicated a lack of SMAD4 on the transcription levels of PARP1 (Supplementary Fig. 2C, D). These findings suggested a contribution of SMAD4 to the attenuation of DNA damage repair, perhaps primarily through its interaction with PARP1, rather than by influencing its transcription.

### The MH1 domain of SMAD4 binds to the DBD domain of PARP1

**Fig. 4:**
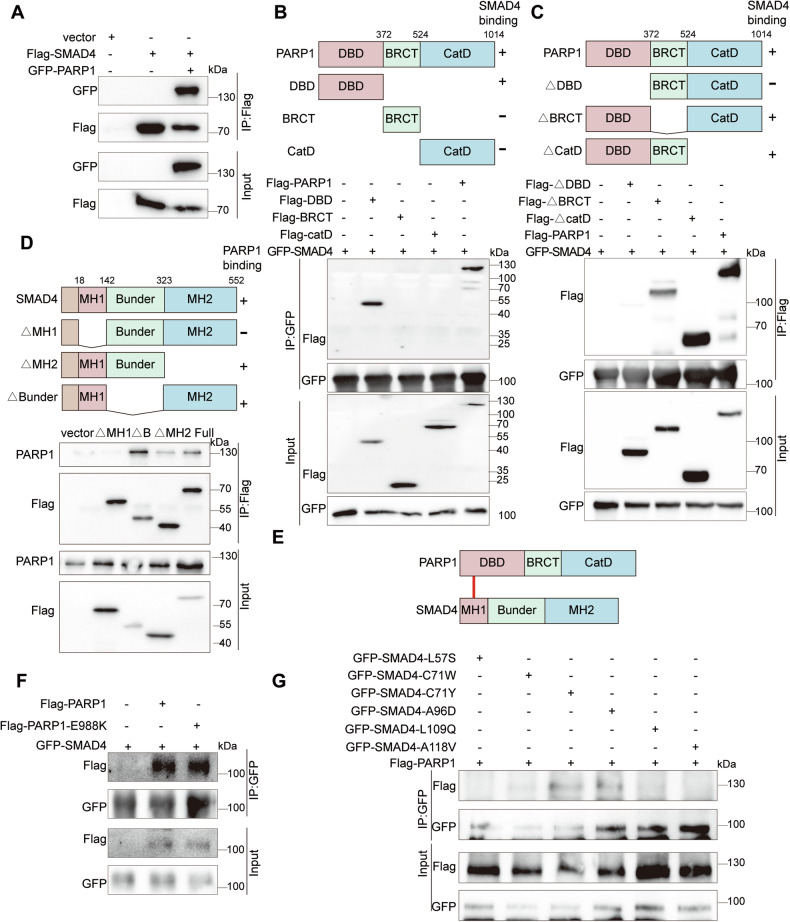
The MH1 domain of SMAD4 binds to the DBD domain of PARP1. **A** Gels showing the interaction of SMAD4 with PARP1. **B** Schematic presentation of PARP1 constructs (WT and mutants), and gels showing the PAPR1 DBD domain bound to SMAD4. **C** Schematic presentation of PARP1 constructs (WT and mutants), and gels showing the PAPR1 construct missing the BRCT domain (△BRCT) and that missing the catalytic domain (△CatD) both bound to SMAD4. **D** Schematic presentation of SMAD4 constructs (WT and mutants), and gels showing △Bunder and △MH2 domains both bound to PARP1. **E** Schematic presentation of the interaction of SMAD4 with PARP1. **F** Gels showing the PAPR1 E988K mutant bound to SMAD4. **G** Gels showing the SMAD4 C71Y and A96D mutants both bound to PARP1.

To determine which domainsmediate the interaction between SMAD4 and PARP1, we conducted a structural domain mapping analysis. Initially, we confirmed the interaction between SMAD4 and PARP1 in HEK293FT cells (Fig. 4A). We then generated various domain constructs of SMAD4 and PARP1 to identify their specific binding sites. Based on our results, the DNA binding domain (DBD) of PARP1, rather than the catalytic domain (CatD) or BRCT domain, was concluded to primarily associate with SMAD4 (Fig. 4B, C). Furthermore, the MH1 domain of SMAD4, rather than the Bunder or MH2 domains, was concluded to be critical for this interaction (Fig. 4D). Overall, our findings confirmed a direct interaction between SMAD4 and PARP1 (Fig. 4E). Given the role of the PARylation modification following recognition of damaged DNA by PARP1, we investigated whether the binding of SMAD4 to PARP1 would be influenced by this modification. Notably, the interaction was found to be unaffected by PARylation, as demonstrated by the PARylation-deficient PARP1-E988K mutant having still bound SMAD4 (Fig. 4F).

In the context of pancreatic cancer, approximately 60% of SMAD4 mutations exhibit loss of heterozygosity, and about 50% involve homozygous deletions or intragenic inactivating mutations [14]. We examined several key mutations in the N-terminal MH1 domain, including L57S, C71W, C71Y, A96D, L109Q, and A118V, based on previous studies and TCGA data [21, 22] (Supplementary Fig. 3A, B). In our examination, the C71Y and A96D mutants retained the ability to bind PARP1, whereas the L57S, L109Q, and A118V mutants did not (Fig. 4G). Thus, we hypothesized that the mutations that led to a failure to bind PARP1 may not interfere with the function of PARP1.

### SMAD4 attenuates PARP1-mediated DNA repair

**Fig. 5:**
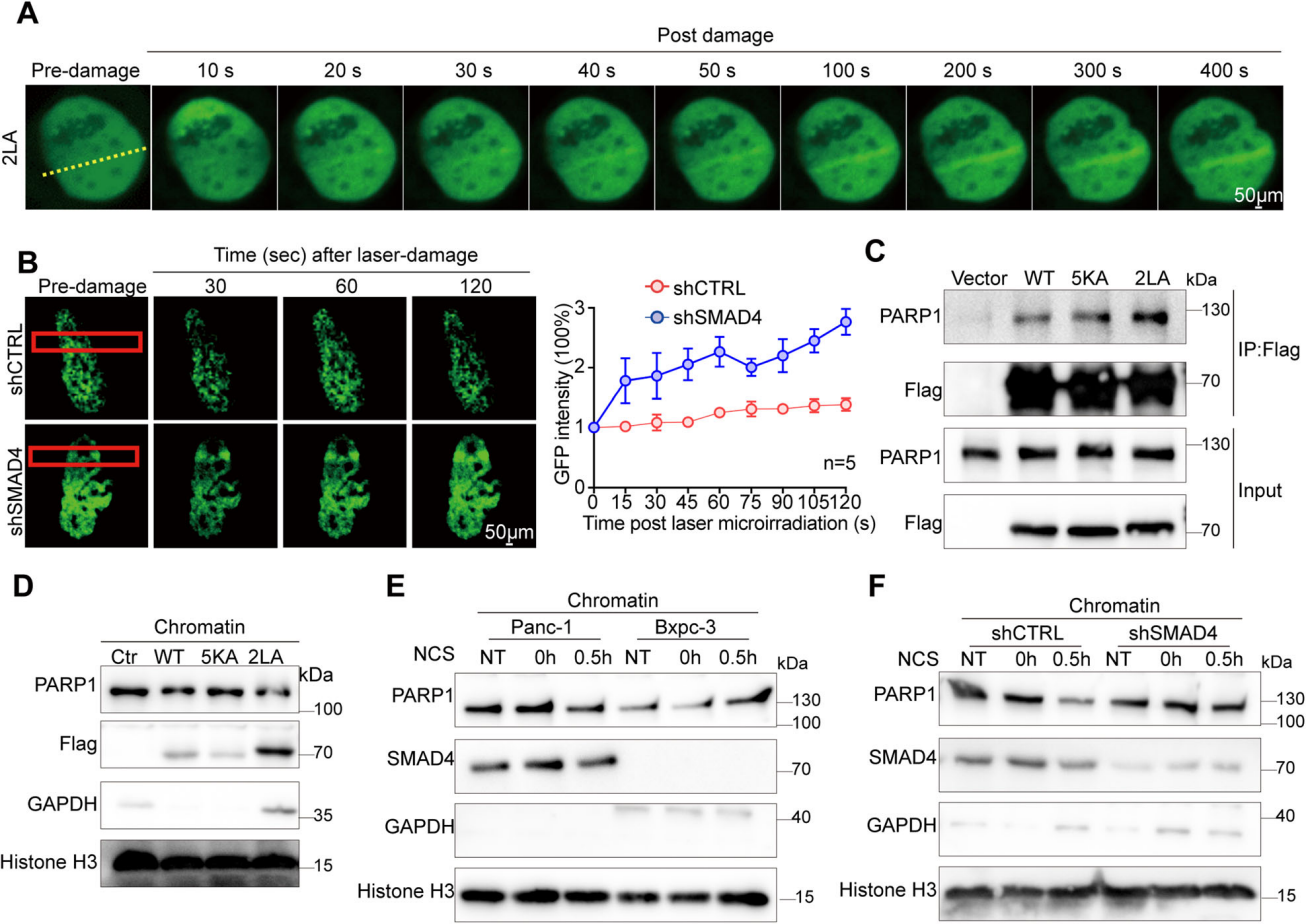
SMAD4 attenuates PARP1-mediated DNA repair. **A** Images showing SMAD4 recruited to DSBs. **B** Images showing SMAD4 having suppressed PARP1 recruitment to DSBs. **C** Gels showing SMAD4 mutants bound to PARP1. **D** Gels showing the SMAD4-2LA mutant having suppressed PARP1 recruitment to chromatin. **E** Gels showing a suppression of PARP1 recruitment to chromatin in SMAD4 overexpressing cells treated with NCS. **F** Gels showing a suppression of PARP1 recruitment to chromatin in shCTRL cells treated with NCS. *P < 0.05, **P < 0.01, and ***P < 0.001 by unpaired Student’s t-test.

To determine whether SMAD4 could impede DNA repair by obstructing access of PARP1 to DNA sites, we transfected HEK293FT cells with nucleus-localizing GFP-tagged SMAD4-2LA. This construct localized strongly at DNA damage tracks **(**Fig. 5A), suggesting recruitment of SMAD4 to DNA damage sites, and implying a potential role for SMAD4 in DNA repair processes. Then, we further assessed the recruitment of GFP-tagged PARP1 to DNA damage sites using laser micro-irradiation in Panc-1 cells with and without SMAD4 knockdown (shSMAD4). The shSMAD4 cells showed more pronounced PARP1 localization to DNA damage tracks than did the control cells **(**Fig. 5B), indicating that in our experiment SMAD4 inhibited DNA repair by affecting PARP1 recruitment. To dissect this function further, we carried out further experiments with the nucleus-localizing SMAD4-2LA and the nucleus-excluded SMAD4-5KA mutants. Co-IP assays in HEK293FT cells revealed that SMAD4-2LA exhibited a stronger interaction with PARP1 than did SMAD4-5KA (Fig. 5C), suggesting an enhanced interaction of SMAD4 interaction with PARP1 resulting from nuclear localization of SMAD4. We then examined the distribution of PARP1 in chromatin and soluble fractions of HEK293FT cells transfected with Flag-tagged SMAD4 and its mutants. Lower levels of PARP1 were detected in the chromatin fractions of cells expressing SMAD4-2LA than in those expressing SMAD4-WT or SMAD4-5KA (Fig. 5D). Finally, we replicated these experiments in PDAC cell lines treated with NCS, which as described above simulates radiotherapy effects: in SMAD4-deficient cells, chromatin-bound PARP1 levels increased following NCS exposure; conversely, cells expressing SMAD4 showed reduced PARP1 levels over time (Fig. 5E, F). Collectively, these data confirmed that SMAD4 modulates DNA repair by limiting PARP1 recruitment to DNA damage sites.

### Combining PARP inhibitor with radiotherapy appears to synergistically decreases the growth of SMAD4-deficient PDAC in vivo and in vitro

**Fig. 6:**
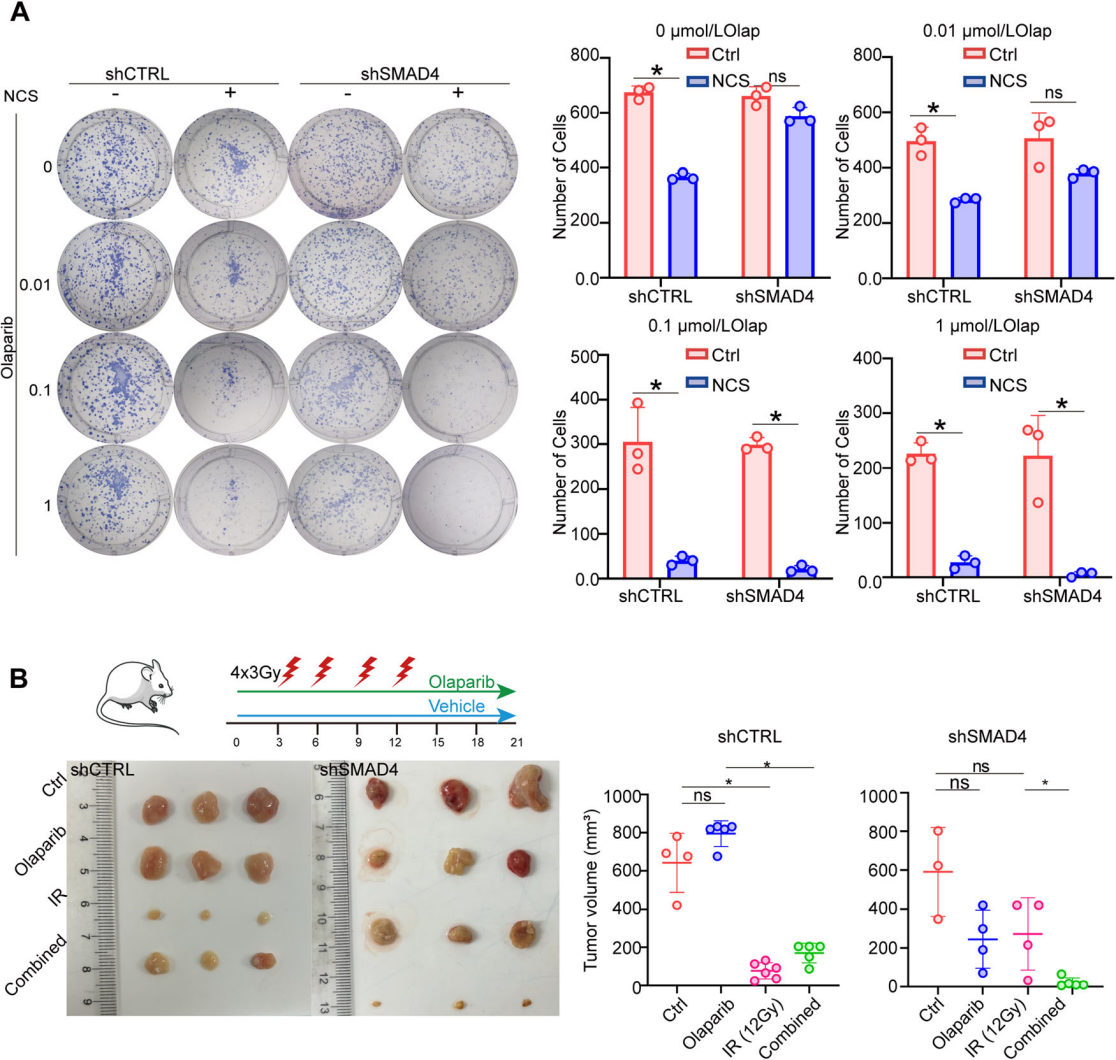
Combining PARP inhibitor with radiotherapy appears to synergistically decreases the growth of SMAD4-deficient PDAC in vivo and in vitro. **A** Clonogenic formation assay to evaluate the cell viability of Panc-1 shCTRL and shSMAD4 cells treated with NCS and olaparib. **B** Results of the analysis of tumor growth in vivo. Nude mice bearing PDAC tumors were orally administered olaparib and radiation, either alone or in combination. *P < 0.05, **P < 0.01, and ***P < 0.001 by unpaired Student’s t-test.

Next, we investigated the therapeutic efficacy of PARP inhibitors as adjuvant drugs during radiotherapy across different SMAD4 statuses in pancreatic cancer. We used olaparib, a PARP inhibitor, for both in vitro and in vivo investigations. Additionally, NCS, the radiomimetic drug, was used for in vitro experimentation. In vitro, we assessed treatment response to various doses of olaparib and/or NCS in clonogenic colony formation assays. The shSMAD4 cells showed enhanced sensitivity to NCS when combined with olaparib (Fig. 6A).

Then, we examined the function of radiotherapy and olaparib on therapeutic responses in subcutaneous tumors (Fig. 6B). Neither the shCTRL group nor the shSMAD4 group showed significant benefit from treatment with only olaparib. Furthermore, in the shSMAD4 group, there was no notable difference between the tumor response to radiotherapy and that to olaparib treatment. However, the shCTRL group exhibited a notable decrease in tumor size after radiotherapy. And, particularly notably, the combination of olaparib and radiotherapy specifically reduced tumor growth in SMAD4-deficient PDAC. Surprisingly, in the SMAD4-proficient group, the addition of olaparib did not enhance the efficacy of radiotherapy.

In summary, these results may suggest that tumors with high SAMD4 expression could be effectively managed via direct radiotherapy. Conversely, SMAD4-deficient tumors could potentially benefit from a combined treatment involving radiotherapy and olaparib.

## Discussion

Radiotherapy is a major therapeutic strategy for both postoperatively, as an adjuvant therapy after surgical resection, and preoperatively, as a neoadjuvant therapy prior to tumor resection of PDAC [23, 24]. However, the development of intrinsic radioresistance in SMAD4-deficient PDAC patients inevitably leads to cancer relapse and a poor prognosis [3, 4]. Our prior research identified SMAD4 deletion in radiotherapy resistance whereby ROS induction and radiation-induced autophagy levels escalated [5]. In our current study, we further demonstrated that SMAD4 deficiency promotes DSB repair in pancreatic cancer caused by ionizing radiation, as evidenced by more quickly decreased expression of γ-H2AX levels.

The mechanism by which PARP1 is involved in DNA single-strand break repair has been widely recognized [25–27]. However, increasing evidence suggests that PARP1 also plays a crucial role in mediating the complex DSB repair pathway in response to severe DNA damage induced by radiotherapy, thereby promoting tumor radioresistance [28–30]. PARP1, as a DNA damage response (DDR) sensor in the nucleus, identifies DNA lesions and activates DDR-associated proteins. Most of the current relevant research focuses on the post-transcriptional modifications affecting PARP1 activity, including PARylation, ubiquitination, and acetylation [31]. For instance, deubiquitinase enzymes, like USP15 and USP1, interacting with PARP1 to ensure its stability and promote DNA repair, genomic stability, and cell growth [32, 33]. Similarly, HECTD3-mediated K63-linked polyubiquitination of PARP1 results in DNA lesions in glioblastoma cells, increasing apoptosis vulnerability [34]. Moreover, acetylation enhances the interaction of PARP1 with MARVELD1, promoting genomic stability in colorectal cancer cells post-DNA damage [35]. However, fewer studies have been conducted on the factors that influence the process of binding of PARP1 to DNA, an important aspect of PARP1 recognition of DNA damage sites. In the present study, we revealed SMAD4 to be a key protein affecting the ability of PARP1 to effect DSB repair by blocking the process of the binding of PARP1 to damaged DNA.

SAMD4 is commonly recognized as a transcription factor that regulates the expression of target genes. Thus, current research on the relationship between SMAD4 and the PARP1 protein is primarily confined to the tumor development process. For instance, PARP1 can attenuate the Smad-specific gene responses and TGF-β-induced epithelial-mesenchymal transition by ADP-Ribosylates SMAD4 [17]. Normally, SMAD4, as a key molecule downstream of TGF- β, enters the nucleus and functions as a transcription factor upon activation of the TGF-β signaling pathway. Our previous research confirmed that PDAC lacking SMAD4 are resistant to radiation5. Mechanistically, the loss of SMAD4 elevates autophagy levels, and autophagy inhibitor reverse this resistance effectively. However, our current study revealed that SMAD4 can be stimulated by radiotherapy to enter the nucleus and bind directly to PARP1 to function as a non-transcription factor. Mechanistically, SMAD4 situated in the nucleus due to radiotherapy can through its MH1 region bind to the DBD region of PARP1, thereby interfering with the efficiency of the binding of PARP1 to damaged DNA, and hence playing a direct regulatory role, which in turns reduces the recruitment of PARP1 to DNA damage sites. Thus, our studies indicated that SMAD4 decreases radioresistance in PDAC through different mechanisms in the cytoplasm and nucleus. Briefly, SMAD4 reduces autophagy in the cytoplasm and inhibits PARP1 in the nucleus.

Considering the strong resistance displayed by SMAD4-deficient patients to the intended effects of radiotherapy, it is particularly important to select appropriate adjuvants to enhance their radiotherapy sensitivity. PARP inhibitors increase DNA damage in BRCA1/2-deficient tumor cells, preventing repair and thereby increasing cell death, a phenomenon called synthetic lethality. Consistent with this mechanism, the maintenance therapy PARP inhibitor olaparib was found in clinical trials to notably prolong progression-free survival of BRCA-deficient PDAC patients [36–38]. However, only 5-10% of pancreatic cancer patients have germline or somatic mutations in the BRCA1 or BRCA2 genes [1, 13], which limits the application of therapies involving PARP1. Our study confirmed that the combination of the PARP inhibitor Olaparib and radiotherapy uniquely decreases the growth of SMAD4-deficient PDAC in vivo and in vitro, perhaps suggesting that using olaparib as an adjuvant in radiotherapy may overcome radioresistance in SAMD4-deficient PDAC patients.

**Fig. 7:**
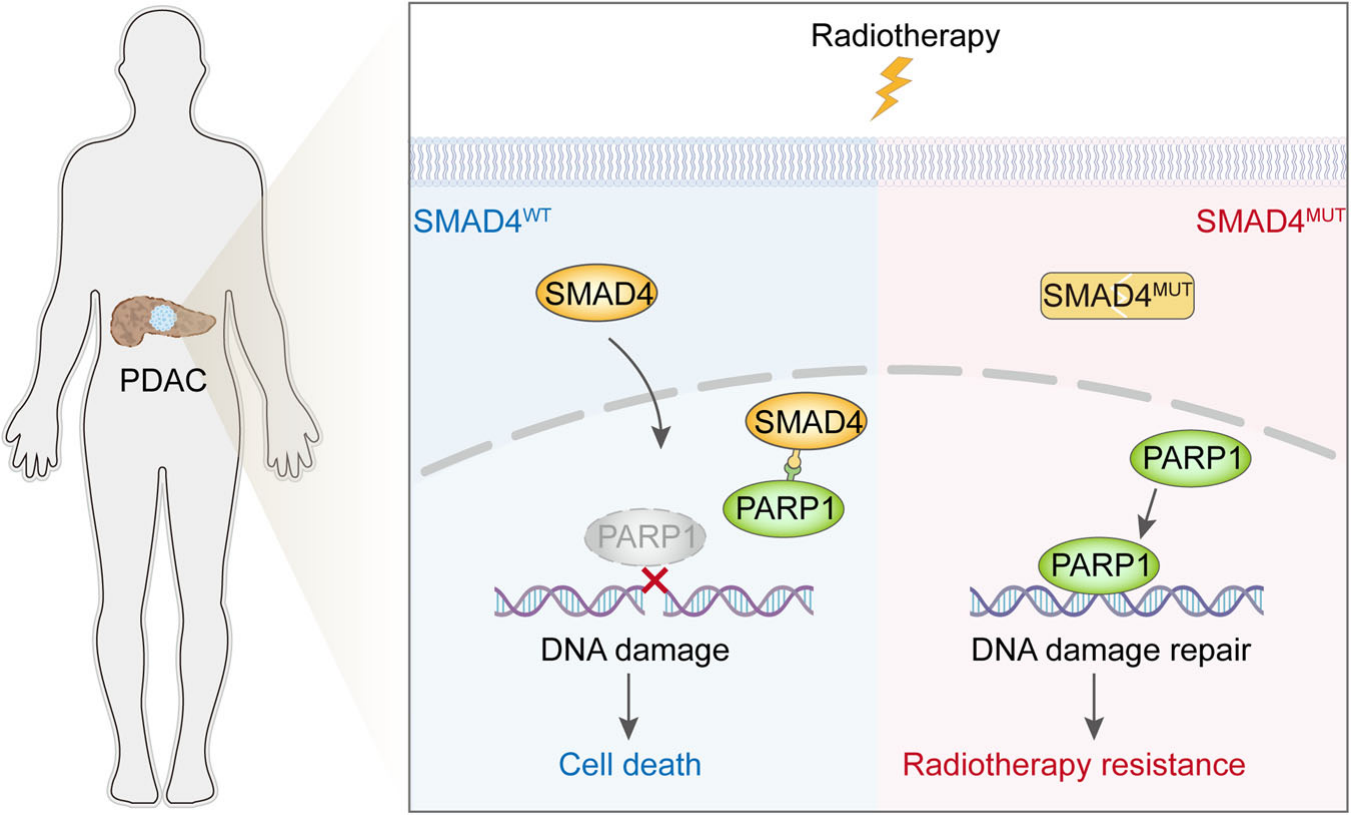
SMAD4 attenuates PARP1-mediated DNA repair and is involved in pancreatic cancer radiotherapy resistance.

In conclusion, our findings revealed a novel molecular mechanism for the SMAD4-PARP1 interaction in DSB repair, in turn uncovering a key mechanism for the development of radioresistance in SMAD4-deficient PDAC patients. Mechanistically, SMAD4 can be translocated to the nucleus in a non-TGF-β-dependent manner by being subjected to radiotherapy, followed by its MH1 domain binding to the PARP1 DBD domain, which is a key region for PARP1 to be able to recognize DNA damage, thereby reducing the recruitment of PARP1 to DNA damage sites. Furthermore, our results showed a synergistic effect of combining the PARP inhibitor olaparib with radiotherapy on the growth of SMAD4-deficient PDAC, which may contribute to determining the optimal treatment strategy for PDAC patients (Fig. 7). In summary, our study elucidated the pivotal role of SMAD4, a gene commonly mutated in PDAC, in attenuating PARP1-mediated DNA repair and contributing to radiotherapy resistance in pancreatic cancer. Nevertheless, further clinical investigations are necessary to evaluate the correlation between SMAD4 status and the survival advantages of radiotherapy in pancreatic cancer patients, which could potentially help guide the administration of targeted therapies in the adjuvant setting based on SMAD4 status.

### Reporting summary

Further information on research design is available in the Nature Research Reporting Summary linked to this article.

## Supplementary information

Supplementary Figure 1-3

Supplementary table

Western blot

Reporting Summary

## Data Availability

The Cancer Genome Atlas (TCGA) data are available through the Genomic Data Commons Data Portal (https://portal.gdc.cancer.gov/). The data that support the findings of this study are available from the corresponding author upon reasonable request.
