# Supplementary material for: SMAD4 Limits PARP1 dependent DNA Repair to Render Pancreatic Cancer Cells Sensitive to Radiotherapy
Source: Cell Death Dis. 2024 Nov 11;15(11):818. doi: 10.1038/s41419-024-07210-7 (PMC11555233; doi:10.1038/s41419-024-07210-7)
Supplement: Supplementary file 1 — Supplementary Figure 1-3 [file 41419_2024_7210_MOESM1_ESM.docx]

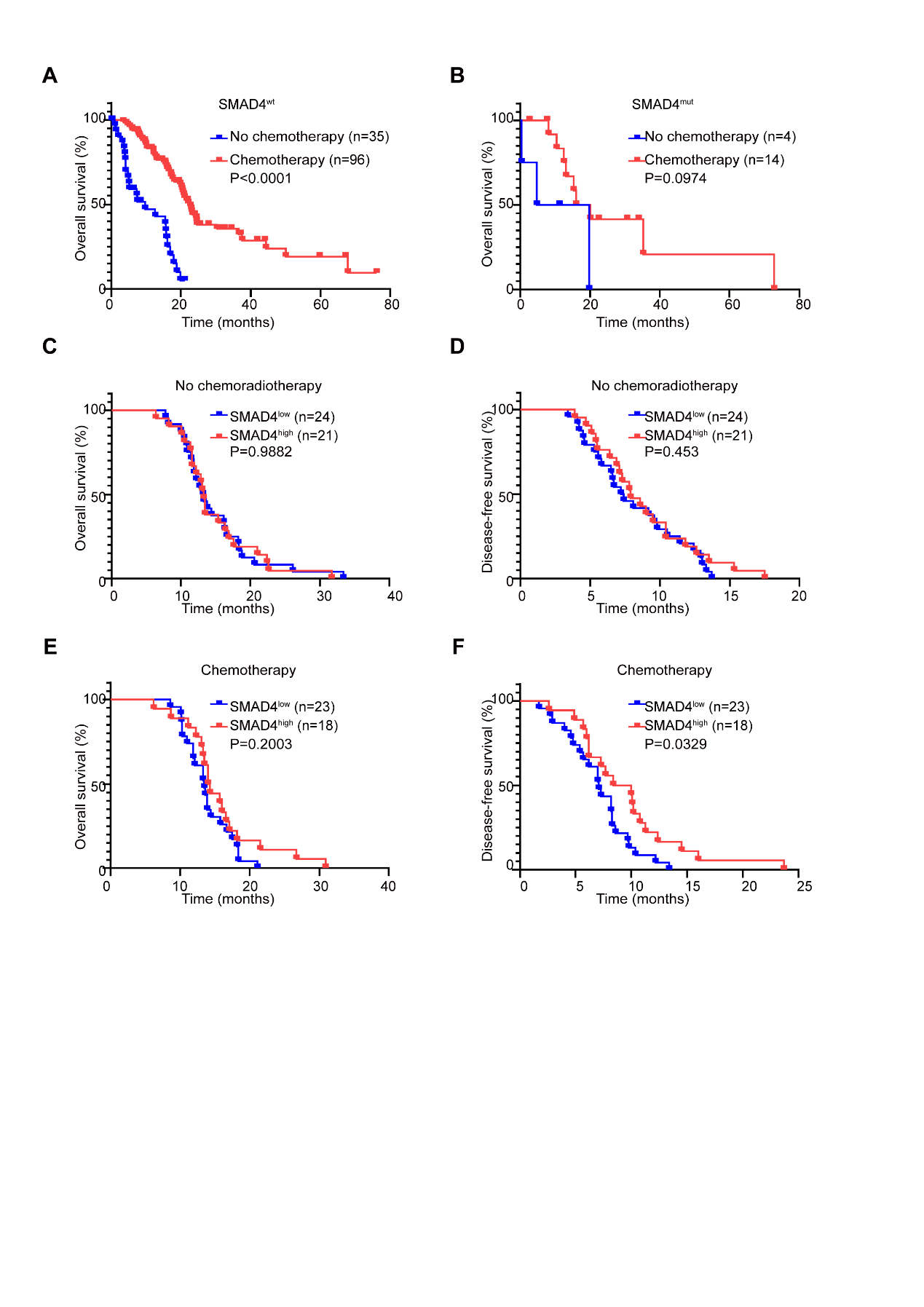


**Figure S1. SMAD4 deficiency endows PDAC with resistance to chemotherapy.**

**A** Plots showing an association of chemotherapy treatment with relatively favorable subsequent OS in SMAD4^wt^ PDAC patients.

**B** Plots suggesting no significant association of chemotherapy treatment with subsequent OS in SMAD4^mut^ PDAC patients.

**C** Plots showing no association of SMAD4 expression level with OS in PDAC patients not receiving chemotherapy.

**D** Plots showing no association of SMAD4 level with DFS in PDAC patients not receiving chemotherapy.

**E** Plots showing no association of SMAD4 level with OS in PDAC patients who had received chemotherapy.

**F** Plots showing a high level of SMAD4 associated with relatively favorable DFS in PDAC patients who had received chemotherapy.


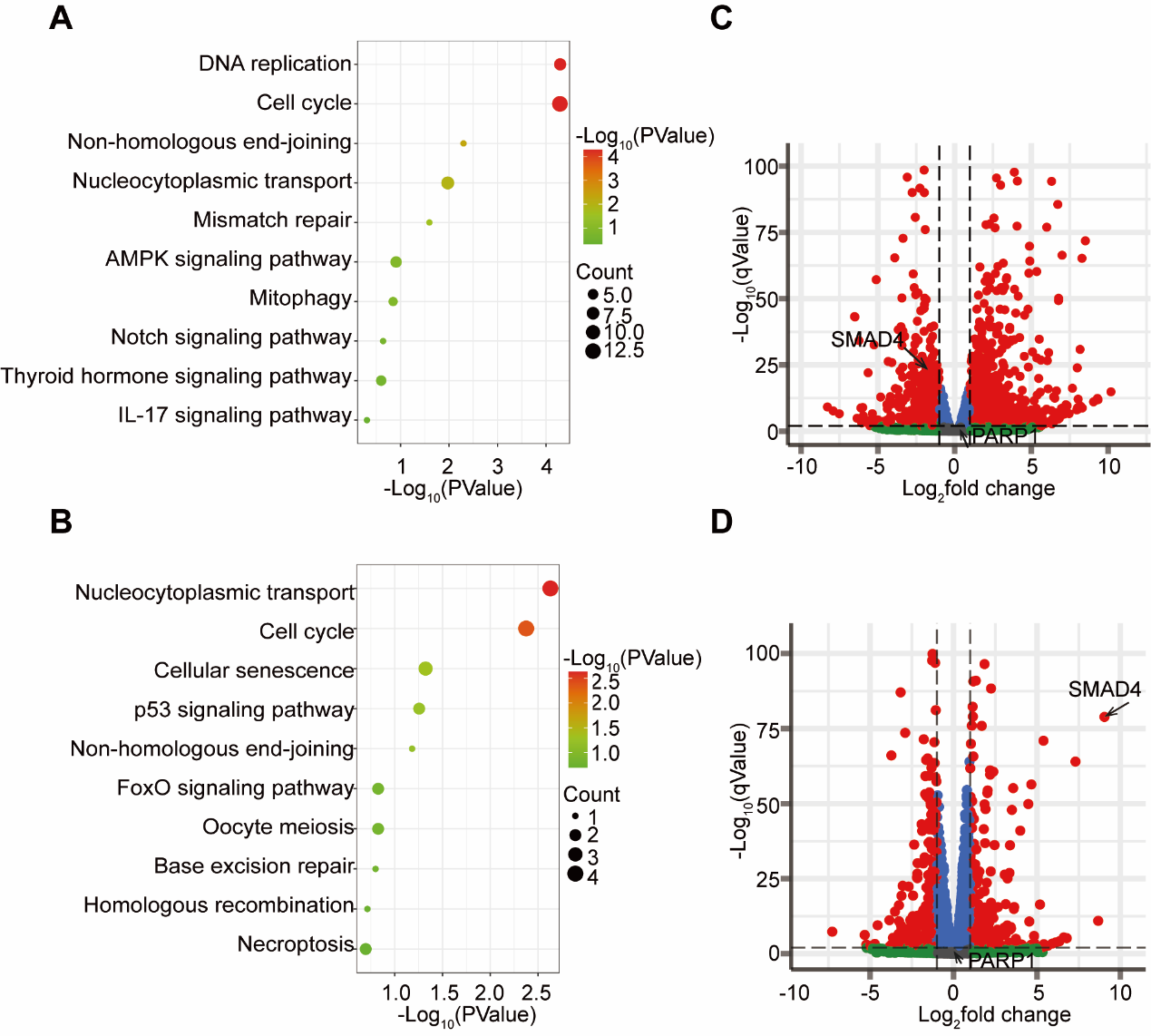


**Figure S2. SMAD4 does not affect the transcription of PARP1.**

**A** KEGG pathway enrichment for higher SMAD4 protein correlations only after irradiation.

**B** KEGG pathway enrichment for higher SMAD4 protein correlations after both control and irradiation.

**C** Volcano plot showing differentially expressed genes (DEGs) in Panc-1 shCTRL and shSMAD4 cells.

**D** Volcano plot showing DEGs in Bxpc-3-vector and Bxpc-3-SMAD4 cells.


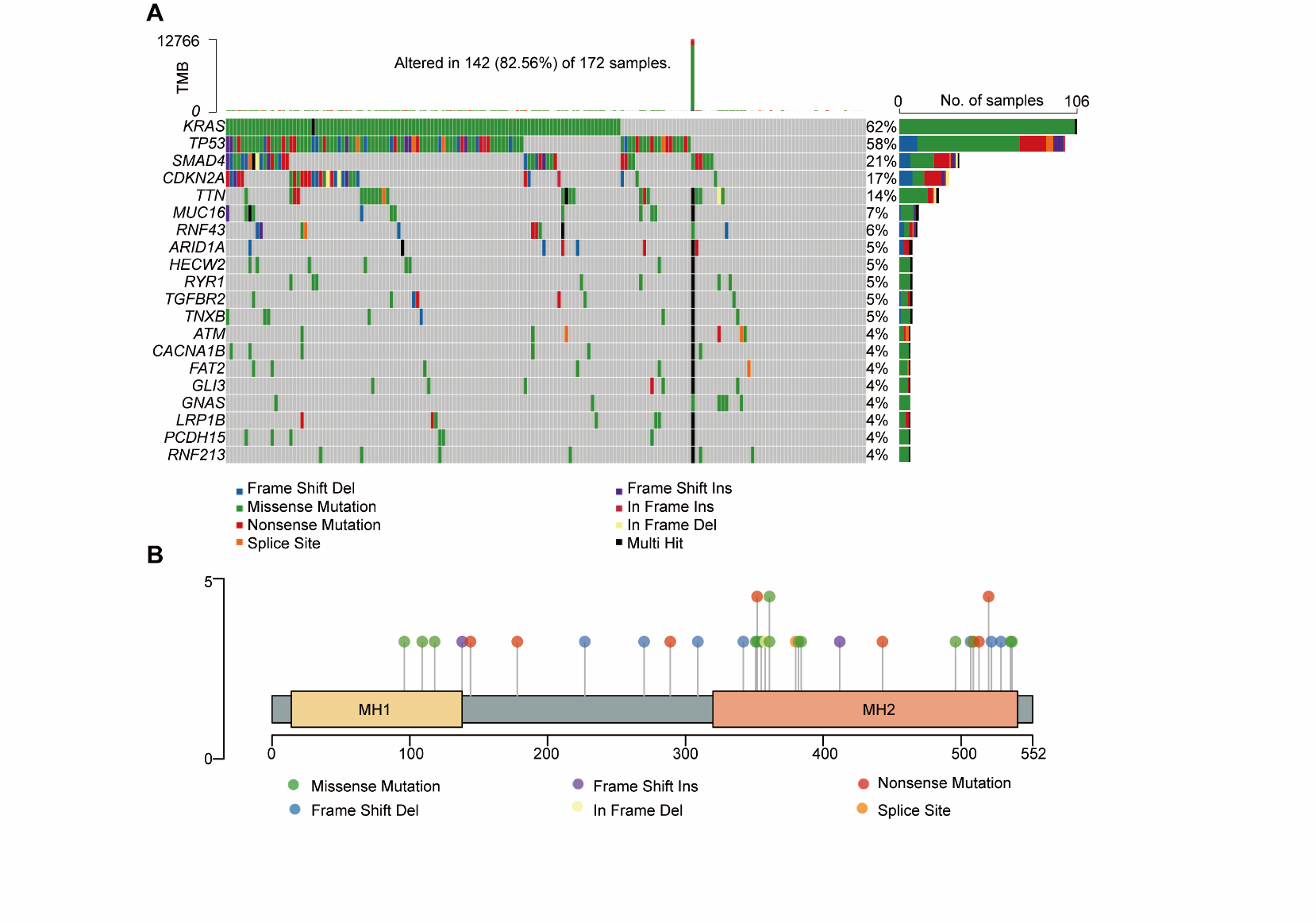


**Figure S3. Common SMAD4 mutations.**

**A** TCGA database analysis showing the frequently mutated genes of PDAC.

**B** TCGA database analysis showing the frequently mutated sites of SMAD4.
